# Screening for major driver oncogene alterations in adenosquamous lung carcinoma using PCR coupled with next-generation and Sanger sequencing methods

**DOI:** 10.1038/srep22297

**Published:** 2016-02-29

**Authors:** Xiaohua Shi, Huanwen Wu, Junliang Lu, Huanli Duan, Xuguang Liu, Zhiyong Liang

**Affiliations:** 1 Peking Union Medical College Hospital, Chinese Academy of Medical Science, Department of Pathology, Beijing 100730, China

## Abstract

We investigated the frequency of major driver oncogenes in lung adenosquamous cell carcinoma (ASC) cases. Frequency of *EGFR, K-Ras, B-Raf, PIK3CA, DDR2, ALK, and PDGFRA* gene mutations was examined in 56 patients using next-generation sequencing, polymerase chain reaction, and Sanger sequencing. Macrodissection or microdissection was performed in 37 cases to separate the adenomatous and squamous components of ASC. The overall mutation rate was 64.29%, including 55.36%, 7.14%, and 1.79% for EGFR, K-Ras, and B-Raf mutations, respectively. PIK3CA mutation was detected in three cases; all involved coexisting EGFR mutations. Of the 37 cases, 34 were convergent in two components, while three showed EGFR mutations in the glandular components and three showed PIK3CA mutations in the squamous components. With respect to EGFR mutations, the number of young female patients, nonsmokers, and those with positive pleural invasion was higher in the mutation-positive group than that in the mutation-negative. K-Ras mutation was significantly associated with smoking. Overall survival in the different EGFR mutation groups differed significantly. The frequency and clinicopathological characteristics of EGFR- and K-Ras-mutated adenosquamous lung carcinoma were similar to that noted in Asian adenocarcinomas patients. The high convergence mutation rate in both adenomatous and squamous components suggests monoclonality in ASC.

Lung cancer is the leading cause of death worldwide and is classified into non-small cell lung cancer (NSCLC) and small-cell lung cancer (SCLC). Adenocarcinoma, squamous-cell carcinoma, and large-cell carcinoma comprise approximately 95% of NSCLCs. Adenosquamous carcinoma (ASC) is a rare variant of NSCLC, accounting for less than 4% of all NSCLC cases^1^. It is defined as a mixed-type tumour, composed of both adenomatous (glandular) and squamous cell components, each comprising at least 10% of the tumour. The prognosis of ASC is believed to be poorer than that of other types of NSCLC, as it is resistant to traditional chemotherapy and more likely to involve early recurrence and metastasis relative to other types of NSCLC^2^,^3^.

In recent years, there has been a dramatic revolution in NSCLC treatment. For example, epidermal growth factor receptor tyrosine kinase inhibitors (EGFR-TKIs) and echinoderm microtubule-associated protein-like 4-anaplastic lymphoma kinase (EML4-ALK) inhibitors are effective and used as first-line treatment agents for patients with adenocarcinoma of the lung who harbour EGFR-TKI-sensitive mutations or EML4-ALK rearrangement^4^,^5^. In addition, pemetrexed and bevacizumab have been approved for the treatment of nonsquamous-cell lung cancer^6^. The poor prognoses of ASC patients have prompted a search for more effective treatments such as targeted therapy. Recently, clinical data have shown that EGFR-TKI is an effective treatment for ASC of the lung^7^.

Besides the success of EGFR and ALK targeted therapy in selected populations, several proto-oncogenes, which coded proteins that were established to play an important role in cellular growth signalling, known as driver oncogenes were reported to be potential targeted genes, they included B-Raf, Kirsten rat sarcoma viral oncogene homolog (K-Ras), phosphatidylinositol-4,5-bisphosphate 3-kinase, catalytic subunit alpha (PIK3CA), discoidin domain receptor tyrosine kinase 2 (DDR2), and platelet-derived growth factor receptor alpha polypeptide (PDGFRA), *et al.* The B-Raf is a proto-oncogene that is a serine/threonine kinase downstream from KRAS in the mitogen-activated protein kinase (MAPK) signalling cascade and can be targeted by Vemurafenib or GSK2118436. K-Ras can initiate cell proliferation through the RAS-dependent kinase cascade and is associated with a worse overall survival (OS) in patients with NSCLC^8^. PIK3CA is a family of lipid kinases that plays an important role in regulating cell growth, proliferation, and survival. DDR2 is a membrane-bound receptor tyrosine kinase that binds to collagen and can regulate proliferation and migration. PDGFRA plays a crucial role in cell proliferation and angiogenesis.

Several reports have been published regarding epidermal growth factor receptor (EGFR) mutation in patients with ASC of the lung but there are few studies concerning other genetic changes in ASC. Moreover, there is an urgent need to elucidate genetic alteration in ASC and develop a precise treatment. In this study, we used polymerase chain reaction (PCR), next-generation (NGS), and Sanger sequencing methods, focusing on the hotspots of seven driver oncogenes, including EGFR, K-Ras, B-Raf, PIK3CA, DDR2, ALK, and PDGFRA, to identify major genetic alterations in patients with ASC of the lung. Macrodissection and laser microdissection methods were employed to separate the two components of ASC of the lung for further analysis. Our study focused mainly on genetic alternations and the implications for origination issues.

## Results

### Patient clinicopathological characteristics

In total, 56 patients were enrolled in the study. Of the 56 patients, 38 were men, 18 were women, 27 were smokers, and 29 were nonsmokers. The median age at diagnosis was 62.39 years (range: 34–80 years). The average tumour size was 4.28 cm (range: 1.5–10 cm). Stages I, II, III, and IV were observed in 16 (28.6%), eight (14.3%), 28 (50%), and four (7.1%) cases, respectively. Subsequent to surgery, 27 of the 54 patients received chemotherapy, 12 were exposed to radiation therapy, and 10 received both types of therapy.

The growth pattern of adenomatous components was classified into lepidic (three), acinar (46), and other types (seven), which included mucinous (one), solid (four), and micropapillary (two) patterns. Differentiation of squamous components was classified as well (eight), moderate (29), and poorly differentiated (19). Lymphovascular invasion was detected in 34 cases, and pleural involvement was identified in 36 cases. Positive node metastasis was present in 39 cases.

In 37 of the 56 cases, two components could be separated via macrodissection or laser microdissection methods, while the remainder were sequenced as mixed tumours because of difficulty in separating them confidently.

### Genetic alteration profiles

**Figure 1:**
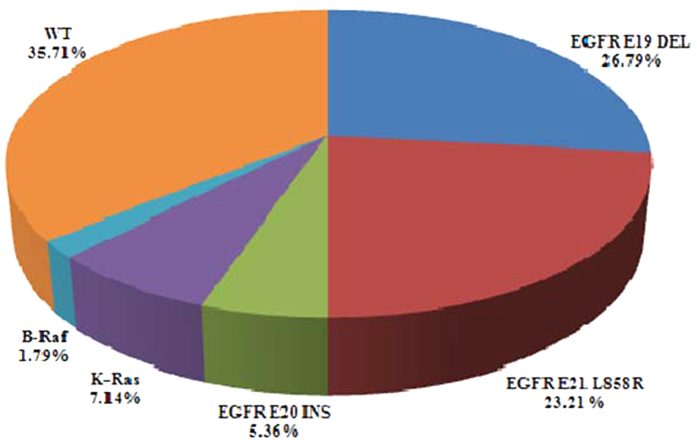
Frequency of mutations in major driver oncogenes in cases of adenosquamous lung carcinoma.

As shown in Fig. 1, the overall mutation rate was 64.29%, with rates of 55.36% (31 cases), 7.14% (four cases), and 1.79% (one case) for mutations in the EGFR, K-Ras, and B-Raf genes. PIK3CA mutation was detected in three cases (5.36%), all of which had coexisting EGFR mutations.

**Figure 2:**
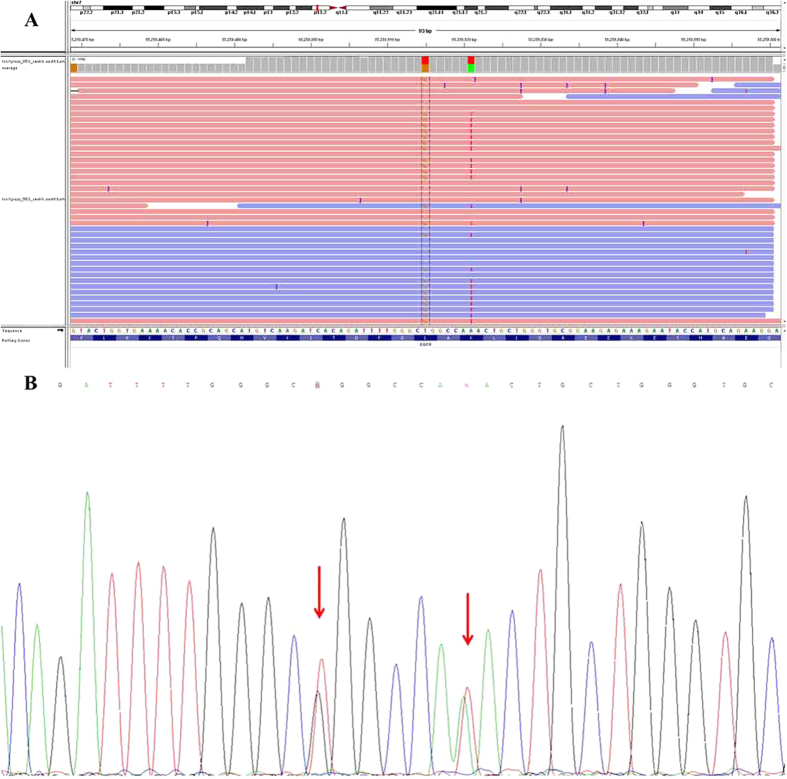
Coexisting EGFR Exon 21 L858R and Exon 21 K860I mutations in one case. (**A**) Next Generation Sequencing (NGS) chromatogram and (**B**) Sanger sequencing chromatogram.

EGFR is the major mutation type in ASC of the lung, with a mutation rate of 55.36%. The most common EGFR mutation was in-frame deletion in exon 19 (15 cases), followed by point mutation (CTG to CGG) in exon 21 at nucleotide 2573, resulting in substitution of leucine by arginine at codon 858 (L858R; 13 cases). Drug-resistant exon 20 insertion mutation (E20-Ins) was observed in three cases. No T790M point mutation was observed. One case showed coexisting EGFR Exon 21 L858R and Exon 21 K860I mutations, as shown in Fig. 2.

K-Ras mutation was found in four cases and mutated exclusively from EGFR. The mutation types were G12C (two cases), G12V (one case), and G12D (one case) in exon 2. B-Raf mutation was identified in one case, with mutation type K601E in exon 15. No somatic mutations were detected in DDR2, ALK, or PDGFRA.

**Figure 3:**
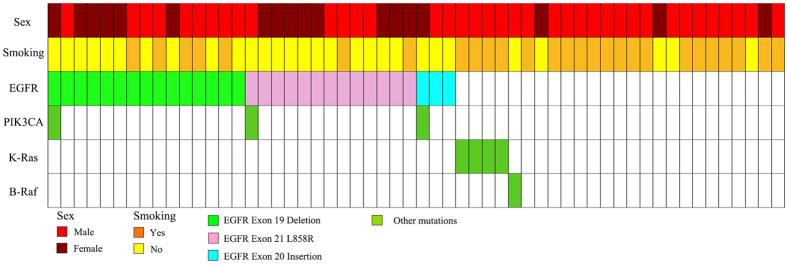
The details of individual cases, with respect to sex, smoking habit, and genetic changes.

For the 37 cases in which the two glandular and squamous components were macrodissected or microdissected and sequenced separately, mutations in the major driver oncogenes, including EGFR (24 cases), K-Ras (one cases), and B-Raf (one case), were identical in both components. The divergent mutation cases are summarized in Table 1. Three cases involved EGFR mutation only in the glandular components of ASC of the lung, while three cases involved PIK3CA mutation only in the squamous components. The details of individual cases, with respect to sex, smoking habit, and genetic changes, are shown in Fig. 3.

### Clinicopathological factors related to genetic alterations

The relationships between EGFR, K-Ras mutations, and clinicopathological factors are summarized in Table 2. With respect to EGFR mutations, the number of young female patients, nonsmokers, and those with positive pleural invasion was higher in the mutation-positive group than that in the mutation-negative group (P = 0.044, 0.004, 0.000, and 0.005, respectively). There was a significant association between K-Ras mutations and smoking (p = 0.048), and all four patients with mutations were smokers.

No significant associations were observed between the groups positive and negative for the EGFR or K-Ras mutations with respect to proportions of predominant subtypes in the adenomatous component, the differentiation of the squamous component, lymphovascular invasion, clinical stages, or tumour sizes. The acinar pattern was the most frequent subtype of the adenomatous component in both the mutation-positive and mutation-negative groups.

### Relationships between clinical outcome and genetic alterations

Survival data were available for 54 patients, as two patients were lost to follow up because of disease recurrence. Overall, 21 (38.89%) patients experienced recurrence or metastasis after an average of 14.37 months (range: 1–54 months). In addition, 15 (28.85%) patients died within an average of 14.67 months after surgery (range: 0–33 months). Upon completion of follow up, 26 patients were alive and without disease.

**Figure 4:**
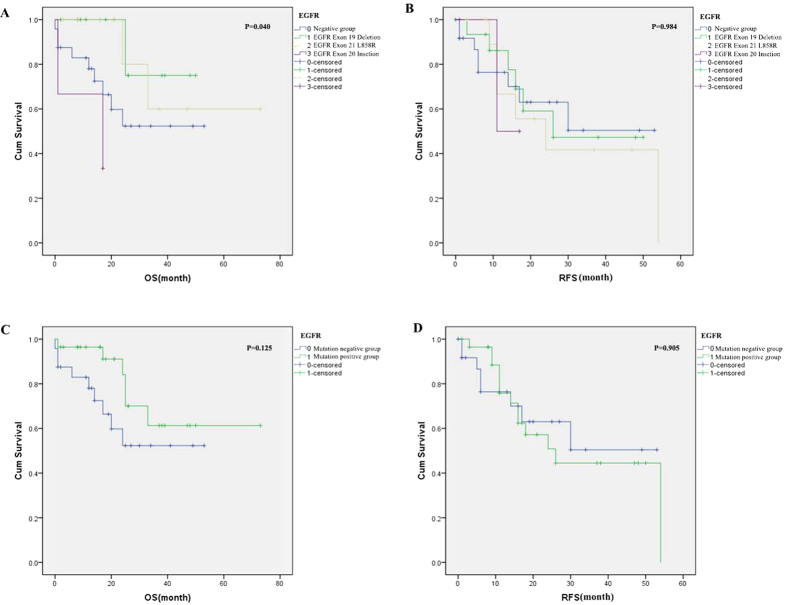
Kaplan–Meier survival curves. (**A**,**B**) OS and RFS in the groups with different EGFR mutations (**C**,**D**) OS and RFS in the groups positive and negative for EGFR mutations.

As shown in Fig. 4, there were significant differences in OS between various the EGFR mutation groups. The group with EGFR mutations in exon 20 exhibited the worst OS (average: 11.7 months), followed by the group with the wild type gene (average: 33.6 months), the group with mutations in exon 19 (average: 43.8 months), and the group with mutations in exon 21 (average: 55.2 months). There were no significant differences in RFS and OS between the K-Ras, EGFR mutated and nonmutated groups.

### Elucidation of Methods

Quantitative PCR was performed successfully for all samples. NGS was performed in 41 cases. The Sanger sequencing method was carried out in 51 samples. All three methods methods were used successfully for examining 41 samples.

The Sanger sequencing method was unable to detect EGFR mutations in two cases, because the mutation rate was below the detection limit of 15% of the whole tumour. NGS was able to identify uncommon mutations (e.g., EGFR exon 21 K860I, EGFR exon 20 insertion, and B-Raf K601E, which cannot be covered by PCR primers). PCR was considered a sensitive and low cost prescreening method, which was used successfully for all study samples and was less limited by the quality or quantity of the samples to a lower extent compared with the other two methods.

## Discussion

ASC is a rare variant of NSCLC, with a poorer prognosis relative to those of the other types of NSCLC^9^. Few studies have evaluated the frequency of major driver oncogene mutations in ASC, with most focusing on EGFR gene mutation^10^,^11^. Results have been inconsistent because of variation in population ethnicity and the use of divergent methods; therefore, further data concerning the frequency of gene alteration in ASC of the lung is required.

In our study, EGFR mutation was the predominant mutation type in ASC of the lung, with a mutation rate similar to that noted in lung adenocarcinoma cases^12^,^13^. Previous large cohort studies have shown a mutation rate ranging from 10.5% to 44% in ASC of the lung^14^,^15^,^16^. EGFR mutation rates may fluctuate because of variation in participant ethnicity, geographic regions, and examination methods. For example, Kang *et al.*^14^ and Jia *et al.*^17^ reported mutation rates of 44% and 39.6%, respectively, in Asian populations; these results are similar to those of the current study. Tochigi *et al.*^18^ and Shu *et al.*^19^ reported mutation rates of 13% and 10.5%, respectively, for EGFR in Western populations; these rates are considerably lower than those observed in Asian people. However, there was a striking discrepancy between studies conducted by Toyooka (3/11, 27.27%)^20^ and Sasaki (4/26, 15.38%)^15^, even though both involved Japanese populations; this inconsistency occurred mainly because of the use of different methods for mutation analysis. Sasaki *et al.* used the more sensitive PCR (amplification-refractory mutation system) method, whereas Toyooka *et al.* employed the less sensitive direct-DNA sequencing method. Our study involved the use of PCR, Sanger, and NGS sequencing methods. NGS and PCR are believed to be much more sensitive than the Sanger sequencing method, which showed a lower detection rate of 1–2%. In our study, the Sanger sequencing method failed to identify EGFR mutations in two patients because the mutation rate was below the method’s detection limits (10% to 15% of the whole tumour). NGS is a promising and comprehensive method, which can be used to detect multiple genes simultaneously, with high sensitivity and accuracy. NGS can amplify DNA to a sufficient level without changing the content. As in PCR, it is not restricted by the primers designed to detect known “hotspot” regions in specific oncogenes. For example, in one patient, the NGS sequencing method revealed both EGFR Exon 21 L858R and Exon 21 K860I mutations; the latter mutation type is a rare variant of the EGFR mutation, which could not be identified with the PCR detection kit because of the lack of availability of PCR primers for this. PCR method seems to be out of date in this era of high throughput technologies, some features like easy-to-interpret data, ability to identify low-frequency mutations, low cost for clinical prescreening and most importantly, low requirement for DNA quality and quantity of the PCR method may out-compete the state-of-the-art NGS method. As was demonstrated by our results, PCR method was successful in all cases, while Sanger Sequencing and NGS method failed in five and 15 samples in the present study, respectively. In the 41 cases where both NGS and PCR methods were successful, the mutations identified were highly consistent, except for those that were not covered by the PCR panel. In addition to the highly sensitive methods, using surgical samples can provide sufficient DNA for detecting EGFR mutations.

In our study, the EGFR mutation was significantly associated with age, sex, smoking, and pleural invasion. Other reports have also confirmed this association; for example, Kang *et al.*^14^ revealed that the EGFR mutation in ASC was more frequent in women and those who had never been smokers. As we know, EGFR-mutated adenocarcinoma patients are likely to be young, women, nonsmokers, and Asian, while squamous cell carcinoma of the lung is believed to have a lower EGFR mutation rate, ranging from 0% to 15%^21^. The EGFR mutation rate detected in our study is consistent with that of adenocarcinoma and considerably higher than that of squamous cell carcinoma of the lung. Based on the above evidence, we could conclude ASC of the lung is another variant of NSCLC, which is more akin to adenocarcinoma, rather than squamous-cell carcinoma, with respect to clinicopathological characteristics and EGFR mutation.

The two predominant types of EGFR mutation in our study were in-frame deletion in exon 19 (15) and exon 21 L858R (13); we further identified three cases of exon 20 insertion mutation, which is thought to be associated with TKI resistance^22^. The EGFR-mutation group had longer OS than the mutation-negative group (53.8 vs 33.6 months); however, the difference between them was nonsignificant (p = 0.125). There were significant differences in OS between the groups with different types of EGFR mutation; the EGFR exon 21 L858R group exhibited the best OS compared with mutation-negative, exon 19 deletion, and exon 20 insertion groups. No previous studies have reported this correlation, which was similar to findings concerning lung adenocarcinoma^23^,^24^,^25^,^26^. Some reports revealed that the EGFR mutation is a prognostic factor in lung adenocarcinoma patients who are treated with chemotherapy^27^. The EGFR mutation group had a longer median survival time than EGFR wild-type patients^28^. Some studies found that EGFR mutations may be associated with the risk of recurrence in cases of curatively resected pulmonary adenocarcinoma^29^, while other studies did not find this association^25^,^30^. We did not observe such a phenomenon between them, perhaps owing to the short follow up duration or the different choice of radiation and chemotherapy.

K-Ras mutation was detected in four of the 56 patients and occurred independently of other genetic changes including EGFR, B-Raf, and PIK3CA mutations. K-Ras mutation tends to appear in patients with a history of smoking; in our study, it was significantly associated with smoking, and all patients with K-Ras mutations were smokers. It is important to identify the K-Ras mutation because of its association with worse clinical outcomes and TKI resistance^31^. There were few data regarding the prognosis of ASC patients with K-Ras mutations. Our results showed that prognoses of ASC patients with K-Ras mutations were not poorer than that of ASC patients with the wild type gene; however, as there were only four mutated cases in our study, more cases should be identified to reach a final conclusion.

Besides the common mutations in the EGFR and K-Ras genes, we identified B-Raf and PIK3CA mutations in cases of ASC of the lung, which could provide data for future studies and targeted therapy. The identification of B-Raf mutations in lung cancer is important, because patients with B-Raf genetic alterations are highly likely to benefit from treatment with B-Raf inhibitors^32^,^33^. Because of the low frequency of B-Raf mutations, there has been no agreement regarding the associations between B-Raf mutations and clinicopathological characteristics, including sex and smoking history, even in lung adenocarcinoma cases. In our study, a B-Raf mutation was detected in a female nonsmoking patient. PIK3CA mutation was detected in three patients (5.4%) with squamous components, which coexisted with an EGFR mutation. Previous studies have shown that PIK3CA mutations occur more frequently in squamous-cell carcinoma relative to adenocarcinoma^34^,^35^ and have always coexisted with the other variants of genetic alteration^36^. Our results further confirmed this phenomenon in ASC of the lung. PIK3CA mutation is thought to be associated with resistance to EGFR TKI therapy. A meta-analysis concluded that adding PIK3CA in the routine gene biomarker analysis is valuable in identifying patients who will experience optimal benefit from EGFR-TKI treatment^37^.

In the 37 cases in which the glandular and squamous components were sequenced separately, the major driver oncogenes (e.g., EGFR [24 cases], K-Ras [one cases], B-Raf [one case] mutations) were identical in both components. These findings could provide a clue to the clonal pathway of ASC. With respect to the histogenesis of mixed-type tumours, monoclonal or polyclonal pathways have been proposed. Monoclonal pathways suggest that this hybrid carcinoma originates from a common progenitor cell, while polyclonal opinion posits that it represents collision tumours originating from separate progenitors. The high frequency of the convergence of genetic mutations in the two components of ASC of the lung supports the monoclonal opinion. Other methods have also been used to confirm that ASC is monoclonal rather than polyclonal (e.g., clonal analysis of the X chromosome)^14^,^38^.

Divergent mutations in the two components were observed in our study. Three cases involved EGFR mutations only in the glandular components of ASC of the lung, while three involved PIK3CA mutations only in the squamous components. EGFR mutations are thought to occur mainly in lung adenocarcinoma. Squamous histology per se is compatible with EGFR/K-Ras mutations. Marchetti *et al.*^39^ and Rekhtman *et al.*^40^ did not observe any mutations in the area encoding the tyrosine kinase domain of EGFR in 454 and 95 Squamous cell carcinoma patients. However, PIK3CA mutation is more common in squamous-cell lung cancer than it is in lung adenocarcinoma. All of the evidence supports the following hypothesis in the evaluation of ASC: Critical events occurring in the progenitor cells of this hybrid tumour cause oncogenesis, such as K-Ras or EGFR mutations, and in the process of tumour development, subsequent differences in the protein expression profiles may promote the acquisition of two different phenotypes.

In conclusion, our results showed that the genotype of ASC is more akin to adenocarcinoma than it is to squamous cell carcinoma. The frequency and clinicopathological characteristics of EGFR and K-Ras mutations in ASC are similar to those of Asian patients with adenocarcinomas. The high convergence rates for identical EGFR, K-Ras, and B-Raf mutations in both adenomatous and squamous components suggest the role of monoclonality in the histogenesis of ASC. Our findings have provided further insight into the molecular biology of ASC carcinoma of the lung and could provide a foundation for the development of a novel treatment strategy for this histological type of lung cancer.

## Methods

### Patient information

Fifty-six patients who had undergone surgery following a final diagnosis of ASC of the lung at Peking Union Medical College Hospital (Beijing, China) between January 2010 and December 2014 were enrolled in this study. All final diagnoses were made according to the morphological findings of hematoxylin and eosin (HE) staining of the tumour sample. For cases involving poorly differentiated or histologically atypical adenocarcinoma or squamous components, additional immunostaining was performed for p63, p40, Napsin A, and thyroid transcription factor 1 to confirm the final results.

Clinical information, including age, sex, smoking habit, clinical stage, treatment methods, relapse-free survival (RFS), and OS were collected from the clinical archives. Clinical stage was determined according to the 7th edition of the American Joint Commission on Cancer Tumor-Node-Metastasis Staging System at the point of surgery. RFS was defined as the time from surgery to relapse or the conclusion of the study. OS was calculated as the time from surgery to death or the conclusion of the research.

For each tumour sample, HE slides were re-reviewed by two experienced pathologists to confirm the final diagnosis and classify the proportion and differentiation of adenomatous and squamous-cell carcinoma components of ASC of the lung. The pathologists had no knowledge of the clinical information concerning the mutation status of the cases during the review. In addition to this information, details concerning the following pathological characteristics were collected: tumour size, lymphovascular invasion, pleural invasion, and node metastasis status. This study was conducted with the approval of the institutional review board of Peking Union Medical College Hospital, and informed consent was obtained from all patients. The methods were carried out in accordance with the approved guidelines.

### Genetic alteration detection

The two components of ASC, which could be separated in HE slides, were dissected by the same pathologist using laser capture microdissection (PixCell II; Arcturus Engineering, Mountain View, CA) or manual macrodissection. DNA was isolated from paraffin-embedded tissues using the QIAamp DNA FFPE Tissue Kit (Qiagen, Hilden, Germany) according to the manufacturer’s instructions. DNA concentration was measured using Nanodrop 2000 (ThermoFisher) and normalized to 20–50 ng/μL.

Mutation profiles for EGFR, K-Ras, PIK3CA, and B-Raf were examined using the Human EGFR Gene Mutation Detection Kit (Real-time Fluorescent PCR), Human K-Ras Gene Mutation Detection Kit, Human PIK3CA Gene Mutation Detection Kit, and Human B-Raf Gene Mutation Detection Kit (Beijing ACCB Biotech, Beijing, China), respectively. The tests covered 63 hotspot mutations including 45 in exons 18, 19, 20, and 21 of EGFR; 12 in exons 2 and 3 of K-Ras; 5 in exons 9 and 20 of PIK3CA; and B-Raf V600E. Quantitative PCR was performed on an Mx3000P PCR instrument (Agilent, Santa Clara, CA, USA) with the following settings: 95 °C for 10 min, 40 cycles of 95 °C for 15 s, and 60 °C for 1 min. Results were interpreted according to the manufacturer’s instructions.

Mutation analysis was performed using the next-generation sequencing method. Libraries were prepared using the NextDay Seq Lung panel (Beijing ACCB Biotech, Beijing, China) on the Ion TorrentTM System (Life Technologies, Carlsbad, CA, USA) according to the manufacturer’s instructions. The genomic regions of EGFR exons 18, 19, 20, and 21, K-Ras exons 2 and 3, PIK3CA exons 9 and 20, and B-Raf exons 11 and 15 were amplified using pooled primer pairs, followed by ligation with adaptors and barcodes. Following purification, libraries were quantified using a Qubit dsDNA HS Assay Kit on a Qubit 2.0 fluorometer (Life Technologies, Carlsbad, CA, USA), diluted to a concentration of 3 ng/mL, and pooled in equal volumes. The library pool was clonally amplified via an emulsion PCR reaction, using Ion Sphere Particles on the OneTouch 2 instrument (Life Technologies) according to the manufacturer’s protocol. Template-positive ion sphere particles were enriched on the Ion OneTouch ES (Life Technologies) according to the manufacturer’s protocol. Following enrichment, sequencing primers and polymerase from the Ion PGM™ Sequencing Supplies 200 v2 Kit (Life Technologies) were added according to the manufacturer’s protocol. The libraries were loaded onto an Ion 318 chip (Life Technologies) and sequenced via an Ion Torrent PGM (Life Technologies) instrument to generate sequencing data. Variants were identified and annotated using the proprietary DanPA bioinformatics pipeline (Beijing ACCB Biotech, Beijing, China).

The Sanger sequencing method was the third method used in the study. Genomic regions of EGFR exons 18, 19, 20, and 21; K-Ras exons 2 and 3; PIK3CA exons 9 and 20, and B-Raf exons 11 and 15 were amplified using DNA samples. The sequencing of each exon was conducted bidirectionally using the primers that were used for the initial amplification reaction and the ABI Prism Big Dye Terminator v 3.1 Cycle Sequencing Kit (Applied Biosystems, Foster City, CA, USA). Sequencing primer extension reactions were analysed using an ABI 3130XL Genetic Analyzer (Applied Biosystems, Foster City, CA, USA) according to the manufacturer’s instructions.

### Statistical analysis

Data were analyzed using the Statistical Package for the Social Sciences (SPSS) Version 20.0.0 Software (SPSS Inc., Chicago, IL). All categorical variables were analyzed by χ2 or Fisher’s exact tests, as appropriate. RFS and OS were calculated via the Kaplan–Meier method. All p values were reported as two-sided, and values <0.05 were considered statistically significant.

## Additional Information

**How to cite this article**: Shi, X. *et al.* Screening for major driver oncogene alterations in adenosquamous lung carcinoma using PCR coupled with next-generation and Sanger sequencing methods. *Sci. Rep.* **6**, 22297; doi: 10.1038/srep22297 (2016).

## Figures and Tables

**Table 1:** Divergent mutation cases in the adenomatous and squamous components of lung ASC.

NO.	Genetic changes	Gender/age	Smoking status	Tumour size(cm)	Node metastasis	Stage	Pleura Invasion	LVI	Status
1	G: EGFR E19 DEL S: WT	Female/58	Never	4	Yes	IIIA	Yes	Yes	Died
2	G: EGFR E21 L858R S: WT	Female/76	Never	5.3	Yes	IIIA	Yes	No	Loss of follow up
3	G: EGFR E19 DEL S: WT	Male/76	Smoker	6	Yes	IIIA	Yes	Yes	Alive with disease
4	G: EGFR E20 INS	Female/62	Never	4	Yes	IIIB	Yes	Yes	Died
	S: EGFR E20 INS + PIK3CA E20 G1050S								
5	G: EGFR E21 L858R	Male/43	Never	3.5	No	IB	No	Yes	Alive without disease
	S: EGFR E21 L858R + PIK3CA E20 H1047L								
6	G: EGFR E19 Del	Female/47	Never	7	Yes	IV	Yes	Yes	Alive with disease
	S: EGFR E19 Del + PIK3CA-E20 E1034G								

ASC: adenosquamous cell carcinoma; S: squamous component; G: glandular component; LVI: lymphovascular invasion; DEL: deletion; INS: insertion; WT, wild type; E19: exon 19; E20: exon 20; E21: exon 21.

**Table 2:** Relationship between EGFR, K-Ras mutations and clinical characteristics of lung ASC patients.

Characteristics	No. of patients (%) Total 56	EGFR Mutation			K-Ras Mutation		
		+	−	P	+	−	P
		31	25		4	52	
Age				0.044			0.353
≥70	17 (30.4%)	6	11		2	15	
<70	39 (69.6%)	25	14		2	37	
Gender				0.004			0.201
Female	18 (32.1%)	15	3		0	18	
Male	38 (67.9%)	16	22		4	34	
Smoking status				0.000			0.048
Smoker	27 (48.2%)	7	20		4	23	
Non-smoker	29 (51.8%)	24	5		0	29	
Tumour size (cm)				0.290			0.582
>3	37 (66.1%)	19	18		3	34	
≤3	19 (33.9%)	12	7		1	18	
Stage				0.166			0.205
Early (I-II)	24 (42.9%)	11	13		3	21	
Advanced (III-IV)	32 (57.1%)	20	12		1	31	
Pleural Invasion				0.005			0.125
Yes	36 (64.3%)	25	11		1	35	
No	20 (35.7%)	6	14		3	17	
Node metastasis				0.519			0.353
Yes	39 (69.6%)	22	17		2	37	
No	17 (30.4%)	9	8		2	15	
Lymphovascular invasion				0.105			0.515
Yes	34 (60.7%)	21	13		2	32	
No	22 (39.3%)	10	12		2	20	
Recurrence*				0.247			0. 492
Yes	21 (38.9%)	13	8		1	20	
No	33 (61.1%)	16	17		3	30	
Death**				0.167			0.674
Yes	15 (28.8%)	6	9		1	14	
No	37 (71.2%)	22	15		3	34	

ASC: adenosquamous cell carcinoma.

Note: *Two patients lost to follow-up; **Four patients lost to follow-up.
